# Lactational Transfer of Long-Chain Perfluorinated Carboxylic Acids in Mice: A Method to Directly Collect Milk and Evaluate Chemical Transferability

**DOI:** 10.3390/toxics8020023

**Published:** 2020-04-01

**Authors:** Yukiko Fujii, Kouji H. Harada, Hatasu Kobayashi, Koichi Haraguchi, Akio Koizumi

**Affiliations:** 1 Department of Health and Environmental Sciences, Kyoto University Graduate School of Medicine, Kyoto 606-8501, Japan; kharada-hes@umin.ac.jp (K.H.H.); hatasuk@doc.medic.mie-u.ac.jp (H.K.); akiokoizumi52@gmail.com (A.K.); 2 Department of Pharmaceutical Sciences, Daiichi University of Pharmacy, Fukuoka 815-8511, Japan; k-haraguti@daiichi-cps.ac.jp; 3 Department of Environmental and Molecular Medicine, Mie University Graduate School of Medicine, Tsu, Mie 514-8507, Japan

**Keywords:** perfluoroalkyl carboxylic acids, lactation, neonatal exposure, intake estimation

## Abstract

Perfluoroalkyl carboxylic acids (PFCAs), such as perfluorooctanoic acid (PFOA, C8), are a group of industrial chemicals that are detected in the serum of people throughout the world. Long-chain PFCAs (C9 to C13) have high lipophilicity, therefore they may have a high transfer rate to breast milk. This study investigated the lactational transfer of PFCAs with carbon chain lengths of 8 to 13 in mice. Lactating dams were given a single intravenous administration of PFCAs (C8 to C13) during the postnatal period (8–13 days after delivery). Milk was collected from the dam 24 h after administration using a milking device built in-house. Plasma was obtained from the dam at the same time as milk collection. The observed milk/plasma (M/P) concentration ratios were 0.32 for C8, 0.30 for C9, 0.17 for C10, 0.21 for C11, 0.32 for C12, and 0.49 for C13. These results indicate that the M/P concentration ratio is not related to the lipophilicity of PFCAs. However, estimated relative daily intake, an indicator of how much PFCA is transferred from dams to pups per body weight, increased with chain length: 4.16 for C8, 8.98 for C9, 9.35 for C10, 9.51 for C11, 10.20 for C12, and 10.49 for C13, which may be related to the lower clearance of long-chain PFCAs. These results indicate the importance of future risk assessment of long-chain PFCAs.

## 1. Introduction

The World Health Organization and academic communities, such as the American Academy of Pediatrics, recommend exclusive breastfeeding for an infant’s first six months [1,2]. Breastfeeding is a standard for infant nutrition, and lowers the risk of many conditions, including otitis media, respiratory tract infections, asthma, obesity, and leukemia [3,4,5,6]. Given the medical advantages of breastfeeding, infant nutrition should be considered a public health issue [2]. However, breastfeeding is also considered a significant exposure route in infants for environmental pollutants [7].

The transfer of chemicals from blood to breastmilk is an important property with regard to breastmilk safety. Chemicals with high lipophilicity are more easily dissolved in fat and transferred into breast milk, because breastmilk is lipid-rich [8]. The milk/plasma (M/P) concentration ratio for a chemical is used as an indicator of its breastmilk transferability [9]. Although the M/P concentration ratio can be partially predicted by physicochemical properties [9], in vivo evaluation is essential because some chemicals have unexpected kinetics.

Perfluoroalkyl carboxylic acids (PFCAs), such as perfluorooctanoic acid (PFOA, C8), have been used as surface tension depressants and additives. PFCAs are present in the environment and have been detected in human serum. Over time, the levels of PFCAs with carbon chain lengths longer than eight (>C8) have increased in the serum of humans from East Asia and Sweden [10,11,12,13]. Perfluorinated compounds may have immunotoxic effects in children [14]. The immune system during the infant period is vulnerable [15,16], therefore PFCA exposure in the infant period is of particular interest, because breastmilk is a substantial dietary source of PFCAs in the infant period [17,18,19]. Estimated logarithm partition coefficients (log K_ow_), an indicator of lipophilicity, increase with the chain length of PFCAs (6.4 for C8, 7.2 for C9, 7.9 for C10, 8.6 for C11, 9.4 for C12, and 10 for C13), as calculated in SciFinder via the advanced chemistry development software V11.02. Long-chain PFCAs (C9 to C13) have higher lipophilicity than C8, and therefore their transfer rate to breastmilk is expected to be higher. However, the secretion ratio of long-chain PFCAs (C9 to C13) has not been fully investigated, despite its toxicological importance.

Previously, we investigated the pharmacokinetics and mass balance of injected PFCAs in male and nonpregnant female mice [20]. The toxicokinetics were significantly different among PFCAs, indicating that lactational transfer could also depend on PFCA chain length. The aim of the present study is to assess the lactational transfer of long-chain PFCAs in an animal model. For this purpose, we conduct a kinetics study of M/P concentration ratios for PFCAs (C8 to C13) in mice. PFCA concentrations in plasma and milk of dams in the postnatal period are evaluated 24 h after intravenous (IV) PFCA administration.

## 2. Materials and Methods 

### 2.1. Materials 

PFOA (C8), perfluorononanoic acid (PFNA, C9), perfluorodecanoic acid (PFDA, C10), perfluoroundecanoic acid (PFUnDA, C11), perfluorododecanoic acid (PFDoDA, C12), tetrabutylammonium hydrogen sulfate, and 11H-perfluoroundecanoic acid were purchased from FUJIFILM Wako Pure Chemical Corporation (Osaka, Japan). Perfluorotridecanoic acid (PFTrDA, C13) was obtained from Sigma-Aldrich (St. Louis, MO, USA). High-performance liquid chromatography (HPLC) grade methanol and methyl tert-butyl ether (MTBE) were obtained from Kanto Chemicals Co. (Tokyo, Japan). A mixture of mass labeled PFCA extraction standards, ^13^C_4_-labeled PFOA, ^13^C_5_-labeled PFNA, ^13^C_2_-labeled PFDA, ^13^C_2_-labeled PFUnDA, and ^13^C_2_-labeled PFDoDA were obtained from Wellington Laboratories Inc. (Guelph, Canada). Benzyl bromide was obtained from the Tokyo Chemical Industry Co. (Tokyo, Japan). All other chemicals used were of the highest purity commercially available.

### 2.2. Animal Handling Procedures

All experimental procedures were approved by the Kyoto University Animal Research Committee (approved No. MedKyo11067) on 30 March 2011. Female FVB/NJcl mice in the postnatal period were used. FVB/NJcl mice were purchased from CLEA Japan, Inc. (Tokyo, Japan) and housed in the Institute of Laboratory Animals, Kyoto University. A standard commercial lab chow diet (F-2, 3.73 kcal/g, Funahashi Farm Corp., Chiba, Japan) was used. All animals were maintained at an ambient temperature of 24 ± 2 °C and 50 ± 10% humidity with a 12-h light/dark cycle (lights on at 7:00 a.m.). Mice were provided with free access to tap water and food. Each PFCA was administered by IV injection into the tail vein. PFCAs were dissolved in ethanol and then prepared with saline (5% ethanol saline). 

On postnatal day (PND) 8 to 13, dams (*n* = 12) were given a single administration of PFCAs through the tail vein (3.13 μmol/kg for each PFCA, injection volume 10 mL/kg). All litters were removed from their dams just before PFCA administration. To observe the PFCA concentrations in dams, milk and blood samples were collected by the following procedure. The dams were anaesthetized with sevoflurane 24 h after administration of PFCAs. Milk was then collected from all dams by aspirating with pulsations (described in Section 2.3). To facilitate milking, a subcutaneous injection of 4.0 U/kg oxytocin was given a few minutes before milking [21]. After aspiration of milk, dams were then placed under sevoflurane anesthesia and euthanized by cervical dislocation. A sample of whole blood was collected and centrifuged (370 g) to isolate plasma. All milk and plasma samples were stored at −20 °C until analyzed.

### 2.3. Milking Device

**Figure 1:**
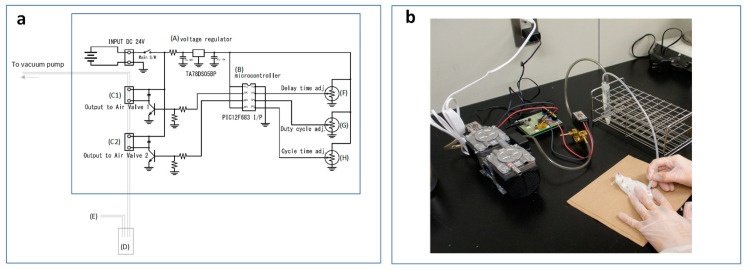
Mouse milking device. (**a**). Schematic circuit diagram of milking device. Voltage regulator (A), a microcontroller (B), two solenoid valves (C1 and C2), a milk-receiving tube (D), a teat cup (E), and three variable resistors (F, G, H). (**b**) Mouse being milked using the milking device.

Milk was collected using an in-house milking device, with modification from the previously described methods for rats [22,23]. A schematic circuit diagram and an overall photograph of the device are shown in Figure 1. 

The milking device consisted of a voltage regulator (TA78DS05BP, TOSHIBA Electronic Devices & Storage Corporation, Tokyo, Japan) (A), a microcontroller (PIC12F683 I/P, Microchip Technology, AZ, USA) (B), two solenoid valves (SMC Corporation, Tokyo, Japan) (C1 and C2), a milk-receiving tube (15 mL centrifuge tube) (D), and a teat cup (Little Leonardo Co., Tokyo, Japan) (E). Vacuum power (6.65 kPa or higher) was obtained from a diaphragm-type dry vacuum pump from ULVAC KIKO, Inc. (Miyazaki, Japan).

To use the milking device, the vacuum pump was started, and three variable resistors (F, G, and H) connected to the two control solenoid valves (C1 and C2) were adjusted to give the optimum pulsation frequency on the teat cup. More detailed information is shown in the Supplementary Materials. Once these were set, it was not necessary to adjust them again. We followed the milking conditions as described by Cox and Mueller (1937), with around 40 pulsations per min. Enough milk (0.5 to 1 mL) was collected from all dams for analysis. 

### 2.4. Determination of PFCAs in Plasma and Milk

Determination of PFCAs was performed by gas chromatography-mass spectrometry (GC-MS, Agilent 6890GC/5973inertMSD) following a previously described method [20,24,25], with slight modification. Briefly, approximately 100 μL milk or 10 μL plasma were used for liquid-liquid extraction. A sample and an internal standard mixture (1 ng each of ^13^C_4_-labeled PFOA, ^13^C_5_-labeled PFNA, ^13^C_2_-labeled PFDA, ^13^C_2_-labeled PFUnDA, and ^13^C_2_-labeled PFDoDA in methanol) were added to the tube. Next, 0.5 mL of 0.5 mol L^−1^ tetrabutylammonium hydrogen sulfate/0.25 mol L^−1^ sodium carbonate buffer (pH 10), 0.5 mL methanol, and 2 mL MTBE were added, and the tube was vortexed for 2 min. The samples were then centrifuged at 9840 g for 5 min and the organic layer was collected. This step was repeated and the organic layers were combined in a clean test tube and then evaporated to dryness under a nitrogen stream. The residue was re-dissolved in 100 µL of a solution of 0.1 mol L^−1^ benzyl bromide in acetone, which contained 10 ng 11H-perfluoroundecanoic acid as an injection standard. The solution was then heated at 60 °C for 1 h to derivatize the PFCAs to benzyl esters. The derivatized PFCAs were analyzed by GC-MS with electron capture negative ionization in the selected ion monitoring mode [24]. The instrumental detection limit was defined as the mass of the analyte producing a peak with a signal-to-noise ratio of three. Milli-Q water was used for the procedural blank control (*n* = 6). For blank levels, the method detection limits (MDLs) were defined by the following equation: MDL = α + 3β, where α is the mean of the blank signals and β is the standard deviation of the blank signals. The MDLs in plasma were 0.02 μmol/L for C8, 0.01 μmol/L for C9 and C10, 0.005 μmol/L for C11, C12 and C13. The MDLs in milk were 0.002 μmol/L for C8, 0.001 μmol/L for C9 and C10, 0.0005 μmol/L for C11, C12 and C13. Thus, the blank levels did not affect the analysis of samples. PFCA levels in serum and milk samples from control mice were less than MDLs.

### 2.5. M/P Concentration Ratio, Estimated Daily Intake (EDI) of Pups, EDI of Dams, and Estimated Relative Daily Intake (ERDI) between Dams and Pups 

#### 2.5.1. M/P Concentration Ratio

The M/P concentration ratio is used as a standard indicator of breastmilk transferability of chemicals [9]. Our previous study showed that PFCAs (C8 to C13) in plasma increase in the 6–12 h after administration and become stable within 24 h [20]. Based on this finding, we collected plasma and milk at 24 h after administration. The M/P concentration ratio was obtained using the following Equation (1): 

(1) $$\begin{array}{c} M/P\;\mathrm{concentration}\;\mathrm{ratio}\;=\\ \;\;\;\frac{\mathrm{Concentration}\;\mathrm{in}\;\mathrm{milk}\;\mathrm{at}\;24\;h\;\mathrm{after}\;\mathrm{administration}\;(µ\mathrm{mol}/L)}{\mathrm{Concentration}\;\mathrm{in}\;\mathrm{plasma}\;\mathrm{at}\;24\;h\;\mathrm{after}\;\mathrm{administration}\;(µ\mathrm{mol}/L)} \end{array}$$

#### 2.5.2. Estimated Daily Intake (EDI) of Pups

The EDI of pups (μmol/kg/day) was obtained using the PFCA concentrations in milk, daily milk consumption, and the calculated bodyweight of pups [26]. All factors used for estimation are summarized in Table 1. Since our study collected milk from dams between PND 8 and 13, we set PND 10 as the evaluation day. The bodyweights of FVB pups were reported as 4.7 g at PND 8, then 6.8 g at PND 13 [27]. Thus, we assumed a body weight gain of 2.1 g between PND 8 and PND 13 was proportional for the period (0.42 g/day). Estimated milk consumption was obtained by doubling the average daily body weight gain (0.84 g milk per day) because 50% of consumed milk weight is assumed to correspond to daily body weight gain [26,28]. The specific weight for breastmilk is 1.017 [29], so daily pup intake of milk was set at 0.85 × 10^−4^ L/day. Body weight for pups at PND 10 was set as 5.5 g based on a previous study [27]. The EDI of pups was calculated using the following Equation (2):

(2) $$\begin{array}{c} \mathrm{EDI}\;\mathrm{of}\;\mathrm{pups}\;(µ\mathrm{mol}/\mathrm{kg}/\mathrm{day})\;=\\ \frac{\mathrm{PFCA}\;\mathrm{concentration}\;\mathrm{in}\;\mathrm{milk}\;(µ\mathrm{mol}/L)\;\times \;\mathrm{estimated}\;\mathrm{milk}\;\mathrm{consumption}\;\mathrm{per}\;\mathrm{day}\;(L/\mathrm{day})}{\mathrm{Pup}\;\mathrm{bodyweight}\;(\mathrm{kg})} \end{array}$$

#### 2.5.3. EDI of Dams

The EDI of dams (μmol/kg/day) was obtained using the plasma PFCA concentrations in this study and the PFCA clearances previously reported by Fujii et al., 2015 [20]. We administered PFCAs to dams in a single dose; therefore, the EDI of dams was converted from the obtained plasma PFCA concentrations [30]. It was assumed that plasma PFCA concentrations 24 h after a single administration reached steady-state and that toxicokinetic parameters were similar between non-lactating and lactating mice. All factors used for the calculation are summarized in Table 1. The EDI of dams was calculated by the following Equation (3):

(3) $$\begin{array}{c} \mathrm{EDI}\;\mathrm{of}\;\mathrm{dams}\;(µ\mathrm{mol}/\mathrm{kg}/\mathrm{day})\;=\\ \;\;\;\;\;\;\mathrm{PFCA}\;\mathrm{concentration}\;\mathrm{in}\;\mathrm{plasma}\;(µ\mathrm{mol}/L)\;\times \;\mathrm{total}\;\mathrm{PFCA}\;\mathrm{clearances}\;(L/\mathrm{kg}/\mathrm{day}). \end{array}$$

A previous study comparing oral and IV administration showed no difference in absorbed dose and 98%–99% of the administered C8 to C13 PFCAs were efficiently absorbed in the gut [20]; therefore, no adjustment was conducted.

#### 2.5.4. Estimated Relative Daily Intake (ERDI) between Dams and Pups

ERDI is an indicator of how much of the dam EDI was transferred to pups via the dam’s milk [31,32]. The ERDI between dams and pups was calculated using the following Equation (4):

(4) $$\begin{array}{c} \mathrm{EDI}\;\mathrm{between}\;\mathrm{dams}\;\mathrm{and}\;\mathrm{pups}\;(\mathrm{ratio})\;=\\ \;\;\;\;\;\;\;\;\;\frac{\mathrm{EDI}\;\mathrm{of}\;\mathrm{pups}\;(µ\mathrm{mol}/\mathrm{kg}/\mathrm{day})}{\mathrm{EDI}\;\mathrm{of}\;\mathrm{dams}\;(µ\mathrm{mol}/\mathrm{kg}/\mathrm{day})} \end{array}$$

### 2.6. Statistical Analysis 

The data are presented as the mean and standard deviation (SD). All statistical analysis was conducted using JMP 13 (SAS Institute Inc., Cary, NC, USA). Statistical significance of the difference in PFCA levels between milk and plasma was assessed using the paired *t*-test (two-sided) with *p* < 0.05 as the acceptance criterion. Statistical significance of the differences in PFCA levels among the different chain lengths (C8 to C13) was assessed using one-way analysis of variance with *p* < 0.05 as the acceptance criterion.

## 3. Results and Discussion

### 3.1. M/P Concentration Ratio of PFCAs

PFCA concentrations in dam plasma and milk at 24 h after PFCA administration are summarized in Table 2. The highest concentrations in plasma were for C8 (13.78 μmol/L), followed by C9 (11.00 μmol/L), C10 (3.43 μmol/L), C11 (2.42 μmol/L), C12 (1.10 μmol/L), and C13 (0.63 μmol/L). This trend was consistent with observations of non-lactating female mice (Fujii et al. 2015). Milk contained measurable levels of PFCAs. The highest concentration in milk was for C8 (4.38 μmol/L), and levels decreased with increasing chain length (C9: 3.30 μmol/L, C10: 0.57 μmol/L, C11: 0.46 μmol/L, C12: 0.29 μmol/L, and C13: 0.23 μmol/L). The concentrations of PFCA in dam milk were significantly lower than those observed in plasma (*p* < 0.05 by paired *t*-test). The observed M/P concentration ratio was 0.32 for C8, 0.30 for C9, 0.17 for C10, 0.21 for C11, 0.32 for C12, and 0.49 for C13. There was a statistically significant difference in M/P ratios among PFCAs (*p* < 0.05, ANOVA). The C8 M/P concentration ratio in the present study was similar to that reported in a previous study (milk/serum concentration ratio was 0.31, when 0.1 mg/kg C8 was administrated on PND 4) [33], supporting the validity of this study. C8 and C9 had comparable M/P ratios to C12 and C13. Considering that longer chain PFCAs have higher lipophilicity, this result revealed that the M/P concentration ratios of PFCAs are not simply related to their lipophilicity_._ Chemicals with an M/P concentration ratio less than 1.0 are classified as low risk [34]. According to this drug safety indicator, all PFCAs were classified as low risk.

The biochemical mechanisms that transfer PFCAs into milk are not well understood; however, the transfer of chemicals to breast milk is affected by physicochemical properties, not only lipophilicity but also protein binding affinity [9]. Chemicals with high plasma-protein binding rates have low transferability to breast milk [8]. PFCAs have a high affinity for protein binding in serum [35,36], which may strongly affect the M/P concentration ratio of them regardless of logP.

### 3.2. Estimated Lactational Transfer from Dams to Pups

The EDI of pups and the ERDI between dams and pups were calculated to evaluate the toxicokinetics of PFCAs (Table 3). The ERDI was calculated from the EDI of pups, which was converted from the PFCA concentration in the dam’s milk, and the EDI of dams, which was converted from the PFCA concentration in the dam’s plasma (see Section 2.5). 

#### 3.2.1. EDI of Pups

The highest amount (µmol/kg/day) was observed for C8 (0.68), followed by C9 (0.51), C10 (0.09), C11 (0.07), C12 (0.05), and C13 (0.04) (Table 3). This study showed that the EDI of pups decreased with increasing chain length. The volume distribution of long-chain PFCAs (C10 to C13) was higher than that of C8 to C9 [20], indicating that long-chain PFCAs (C10 to C13) are more highly distributed in tissue than in plasma. Thus, concentrations of C10 to C13 in breastmilk are lower than those of C8 to C9, although the M/P concentration ratio is similar among C8 to C13.

#### 3.2.2. EDI of Dams and ERDI between Dams and Pups

The EDI of dams (µmol/kg/day) was 0.163 for C8, 0.056 for C9, 0.010 for C10, 0.008 for C11, 0.005 for C12, and 0.005 for C13 (Table 3). The ERDI between dams and pups (EDI of pups/EDI of dams) was 4.16 for C8, 8.98 for C9, 9.35 for C10, 9.51 for C11, 10.20 for C12, and 10.49 for C13 (Table 3). Hale (2010) categorized drugs with a ERDI of less than 0.1 and rated them as generally safe [31]. According to this drug safety indicator, all PFCAs were classified as high risk. The ERDIs of PFCAs increased with increasing chain length, which may be mostly related to the lower clearance of long-chain PFCAs [20]. This result indicates the importance of future risk assessment for long-chain PFCAs.

### 3.3. Limitations of this Study

There are several limitations of this study that should be noted in its relevance to assessment of human exposure. Human mammary glands express organic anion transporters [37] and PFOA and other PFCAs are substrates for these transporters [38,39,40]. There are some species differences in substance selectivity between mouse and human organic anion transporters [41]. These species differences should be investigated in future studies. Second, during the lactation period, the M/P concentration ratio and ERDI may vary between colostrum and mature milk.

## 4. Conclusions

This study reported the secretion profile (plasma to milk) of PFCAs with six different carbon chain lengths (C8 to C13) in mice. The observed M/P concentration ratios were lowest for C10 (0.17) and highest for C13 (0.49). This indicates that the secretion ratio is not directly related to the estimated log K_ow_ of PFCAs. However, all PFCAs had high ERDI between dams and pups (4<), indicating the importance of future risk assessment of long-chain PFCAs. The animal experimentation method used in this study can be used to investigate environmental pollutants in infants.

## Figures and Tables

**Table 1:** Factors and values used to estimate doses to pups via lactation, and relative doses to pups from dams.

Factor	Value	Reference
(A) PFCA concentrations in milk (C8–C13)	Shown in Table 2 (μmol/L)	the present study
(B) Body weight of FVB mice at PND 8	4.7 g	Eshraghi et al., 2016 [27]
(C) Body weight of FVB mice at PND 13	6.8 g	Eshraghi et al., 2016 [27]
(D) Body weight gain between PND 8 and PND 13	2.1 g	(C)–(B)
(E) Average daily body weight gain	0.42 g/day	(D)/5
(F) Estimated milk consumption weight per day	0.84 g/day	(E) × 2 (Grigor and Thompson, 1987 [28], Goerge et al., 2010 [26])
(G) Estimated milk consumption volume per day	8.5 × 10^−4^ L/day	(F) × 1.017 (Breastmilk specific weight, g/L) × 10^−3^ (Suzuki et al., 2004 [29])
(H) Body weight of pups at PND 10	5.5g	(B) + (E) × 2
**(I) EDI of pups**	Shown in Table 3 (μmol/kg/day)	(A) × (G)/(H) …Equation (2)
(J) PFCA concentrations in plasma (C8–C13)	Shown in Table 2 (μmol/L)	the present study
(K) Total PFCA clearances (C8–C13)	C8; 0.012, C9; 0.005, C10; 0.003, C11; 0.003, C12; 0.005, C13; 0.007 (L/kg/day)	Fujii et al., 2015 [20]
**(L) EDI of dams**	Shown in Table 3 (μmol/kg/day)	(J) × (K) …Equation (3)
**(M) ERDI between dams and pups**	Shown in Table 3 (ratio)	(I)/(L) …Equation (4)

PFCA: Perfluoroalkyl carboxylic acids. PND: postnatal day. EDI: Estimated daily intake. ERDI: Estimated relative daily intake. Bold factors are shown in Table 3.

**Table 2:** Perfluoroalkyl carboxylic acids (PFCA) concentrations in milk and plasma in lactating mice, 24 h after a single IV administration (3.13 µmol/kg).

		PFOA	PFNA	PFDA	PFUnDA	PFDoDA	PFTrDA
		(C8)	(C9)	(C10)	(C11)	(C12)	(C13)
Milk	µmol/L	4.38 (1.15) *	3.30 (2.15) *	0.57 (0.20) *	0.46 (0.19) *	0.29 (0.10) *	0.23 (0.07) *
Plasma	µmol/L	13.78 (2.21)	11.00 (5.46)	3.43 (0.75)	2.42 (1.63)	1.10 (0.53)	0.63 (0.39)
Milk/Plasma ^a^	ratio	0.32 (0.07)	0.30 (0.10)	0.17 (0.07)	0.21 (0.07)	0.32 (0.15)	0.49 (0.27)

The data are presented as the mean (SD). * *p* < 0.05 versus plasma concentration (paired *t*-test). ^a^ See Equation (1) in Section 2.5.1.

**Table 3:** Estimated lactational transfer from dams to pups.

		PFOA	PFNA	PFDA	PFUnDA	PFDoDA	PFTrDA
		(C8)	(C9)	(C10)	(C11)	(C12)	(C13)
EDI of pups ^a^	µmol/kg/day	0.68 (0.18)	0.51 (0.33)	0.09 (0.03)	0.07 (0.03)	0.05 (0.02)	0.04 (0.01)
EDI of dams ^b^	µmol/kg/day	0.163 (0.025)	0.056 (0.027)	0.010 (0.002)	0.008 (0.005)	0.005 (0.002)	0.005 (0.003)
ERDI between dams and pups ^c^	ratio	4.16 (0.87)	8.98 (3.15)	9.35 (3.84)	9.51 (2.96)	10.20 (4.95)	10.49 (5.87)

The data are presented as the mean (SD). EDI: Estimated daily intake. ERDI: Estimated relative daily intake. ^a^ See Equation (2) in Section 2.5.2. ^b^ See Equation (3) in Section 2.5.3. ^c^ See Equation (4) in Section 2.5.4.

## Supplementary Materials

The following are available online at https://www.mdpi.com/2305-6304/8/2/23/s1, Figure. S1. Movement of two solenoid valves (C1 and C2) in the milking device.
