# Supplementary material for: Lactational Transfer of Long-Chain Perfluorinated Carboxylic Acids in Mice: A Method to Directly Collect Milk and Evaluate Chemical Transferability
Source: Toxics. 2020 Apr 1;8(2):23. doi: 10.3390/toxics8020023 (PMC7356610; doi:10.3390/toxics8020023)
Supplement: Supplementary file 1 [file toxics-08-00023-s001.pdf]

Supplementary Materials: Lactational Transfer of Long-Chain Perfluorinated Carboxylic Acids in Mice: A Method to Directly Collect Milk and Evaluate Chemical Transferability

Yukiko Fujii, Kouji H Harada, Hatasu Kobayashi, Koichi Haraguchi and Akio Koizumi

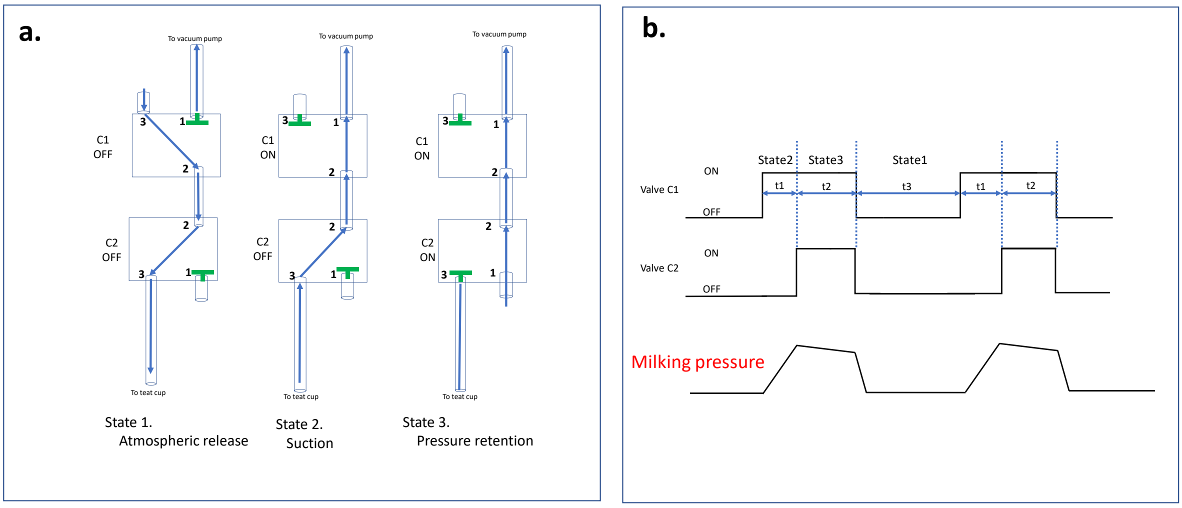

Figure S1. Movement of two solenoid valves (C1 and C2) in the milking device. **a**. Mechanism of pulsation. When valves 1 and 2 are off, the teat cup is released to the atmosphere through ports 3 and 2 of valve C2 and ports 2 and 3 of valve C1 (State 1). When valve C1 is turned on, ports 1 and 2 of valve C1 are connected, and the vacuum pump and the teat cup are connected through ports 1 and 2 of valve C1 and ports 2 and 3 of valve C2 for suction (State 2). When valve C2 is turned on after the time t1, port 3 of valve C2 is shut off, and the negative pressure is maintained (State 3). **b**. Time control of switching with two solenoid valves. The time to open and close the solenoid valves (t1, t2 and t3) is set according to the variable resistor values (F, G, H in Figure. 1).

The two solenoid valves (C1 and C2 in Figure 1) were controlled to create pulsation on the teat cup. As shown in Figure S1, each solenoid valve had ports 1, 2, and 3. When the solenoid is not energized (off), port 1 is closed and ports 2 and 3 are connected. The times to open and close the two solenoid valves (t1, t2, and t3) are shown in Figure S1. When the solenoid is energized (on), ports 1 and 2 are connected and port 3 is closed. Port 1 of valve C1 is connected to the vacuum pump by a tube. Port 2 of valve C1 and port 2 of valve C2 are connected by a tube. Port 3 of valve 2 is connected to the teat cup by a tube. When valves 1 and 2 are off, the teat cup is released to the atmosphere through ports 3 and 2 of valve C2 and ports 2 and 3 of valve C1 (State 1). When valve C1 is turned on, ports 1 and 2 of valve C1 are connected, and the vacuum pump and the teat cup are connected through ports 1 and 2 of valve C1 and ports 2 and 3 of valve C2 for suction (State 2). When valve C2 is turned on after time t1, port 3 of valve C2 is shut off, so that the suction port is closed, and negative pressure is maintained (State 3). When valves C1 and C2 are both turned off at the same time after t2, the state returns to state 1, and the negative pressure of the teat cup returns to atmospheric pressure. This state is maintained for time t3. This is one cycle from state 1 to state 3. The times to open and close the solenoid valves (t1, t2 and t3) are set according to the variable resistor values (F, G, and H, respectively) (Figure 1). To control the volume, we installed an in-house program written in C language in the microcontroller (B) (Figure 1). The program can control pulsation between 30 and 120 per min. The milking program written in C language was as follows;

```
#include <12F683.h>

#FUSES WDT                //Watch Dog Timer
#FUSES INTRC_IO           //Internal RC Osc, no CLKOUT
#FUSES PUT                //Power Up Timer
#FUSES MCLR               //Master Clear pin enabled
#FUSES NOPROTECT          //Code not protected from reading
#FUSES NOCPD              //No EE protection
#FUSES BROWNOUT           //Reset when brownout detected
#FUSES IESO               //Internal External Switch Over mode enabled
#FUSES FCMEN              //Fail-safe clock monitor enabled

#use delay(int=8000000,RESTART_WDT)

signed long t1,t2,t3,d1,d2,d0;

void main()
{

   setup_timer_0(RTCC_INTERNAL|RTCC_DIV_1);
   setup_wdt(WDT_144MS|WDT_DIV_16);

   setup_oscillator(OSC_8MHZ);
   setup_adc(adc_clock_internal);
    setup_adc_ports(san0);
    setup_adc_ports(san1);
    setup_adc_ports(san2);
   while (1)
   {
       set_adc_channel(0);
       delay_us(100);
       d0=read_adc();
       set_adc_channel(1);
       delay_us(100);
       d1=read_adc();
       set_adc_channel(2);
       delay_us(100);
       d2=read_adc();
       t1=d0*2;
       t2=d1*4-t1;
       if(t2<0)
         t2=0;
```

```
    t3=d2*6+500-d1*4;
    if(t3<0)
      t3=0;
    output_high(pin_a5);
    delay_ms(t1);
    output_high(pin_a4);
    delay_ms(t2);
    output_low(pin_a5);
    output_low(pin_a4);
    delay_ms(t3);
   }


}
```
